# A Systematic Review of Methods to Measure Adherence to Oral Anticancer Medications in African Women With Breast Cancer at Initiation, Implementation, and Discontinuation of Therapy

**DOI:** 10.1155/ijbc/5293415

**Published:** 2026-02-27

**Authors:** Deborah Obehi Onwusah, Tafadzwa Mindu, Moses John Chimbari, Elizabeth Bolanle Ojewole

**Affiliations:** ^1^ Discipline of Pharmaceutical Sciences, School of Health Sciences, College of Health Sciences, University of KwaZulu-Natal, Durban, KwaZulu-Natal Province, South Africa, ukzn.ac.za; ^2^ Discipline of Public Health Medicine, School of Nursing and Public Health, College of Health Sciences, University of KwaZulu-Natal, Durban, KwaZulu-Natal Province, South Africa, ukzn.ac.za

**Keywords:** breast cancer, discontinuation, implementation, initiation, measure, medication adherence, oral antineoplastic agents

## Abstract

**Background:**

This systematic review was aimed at assessing methods used to measure oral anticancer medication (OAM) adherence at its three phases (initiation, implementation, and discontinuation) in women with breast cancer (BC) in Africa.

**Methods:**

This review followed the Joanna Briggs Institute guidelines. Four databases were searched from 1990 to 2025 using keywords representing medication adherence, oral anticancer agents, breast cancer, measures, women, and Africa. The reporting followed the updated PRISMA guidelines.

**Results:**

All 13 studies (100%) reviewed assessed OAM adherence at the implementation phase, four studies (30.8%) at the discontinuation phase, and one study (7.7%) at the initiation phase. Persistence was also assessed in four studies (30.8%). Prescription refill records were used to measure adherence at the initiation phase. Drug assays in blood, prescription refills, pill counts, medical records reviews, and self‐report measures were used during the implementation phase. Prescription refills, medical records, and self‐report measures were used at the discontinuation phase. Overall, self‐report measures (46.2%; *n* = 6) were the most frequently used during the implementation phase. However, most studies that employed these measures did not report the psychometric properties, including reliability and validity.

**Conclusions:**

The implementation phase was the most commonly assessed. Initiation and discontinuation phases were relatively less assessed. Self‐report measures were the most frequently used, but the reliability and validity of these measures are lacking, hence limiting the evidence to guide practice. Thus, measures with sound psychometric properties, including reliability and validity, that can assess OAM adherence across its three phases are needed to improve adherence measurement and patient outcomes, particularly in Africa.

## 1. Introduction

Breast cancer (BC) is a growing public health problem and the most common cancer among women worldwide, including in Africa. Globally, BC constitutes about 11.6% of all cancer cases and 6.9% of all cancer deaths [1, 2]. In Africa, several countries, particularly in sub‐Saharan Africa, are among those with the highest BC mortality, revealing inadequate health facilities and services [1, 2]. BC negatively impacts patients′ health, including the physical, mental, psychological, socioeconomic, and financial well‐being of patients and their caregivers [3–8]. A substantial increase in the use of oral anticancer medications (OAMs) comprising endocrine and nonendocrine therapy for cancer, including BC, has been observed in the last few decades [9–11]. Oral endocrine therapy (OET) or hormonal therapy (e.g., tamoxifen and letrozole) is prescribed for patients with hormone receptor–positive (HR+) BC, including neoadjuvant and adjuvant therapy for early‐stage BC and palliative therapy for advanced BC [9]. OET is typically prescribed for at least 5 years, reduces the risk of recurrence by almost half, minimizes the risk of developing contralateral new primary BC, and reduces mortality rates by about one‐third in patients with BC [9, 12, 13]. Oral nonendocrine therapy, including cytotoxic chemotherapy and targeted therapy, has demonstrated improved clinical benefits in BC patients. Cytotoxic chemotherapy (e.g., capecitabine) is prescribed for patients with hormone receptor–negative (HR−) BC and can prolong the survival of patients. Targeted therapy (e.g., trastuzumab, pertuzumab, and lapatinib) is prescribed for patients with HER2‐positive BC, given in combination with chemotherapy, and significantly improves the prognosis of HER2‐positive BC [9, 14].

Adherence signifies “the extent or level of conforming to the recommendations regarding the day‐to‐day treatment by the provider regarding the timing, dosage, and frequency,” and persistence refers to “the duration from the initiation of the medication to discontinuation of therapy” [15]. Expanding this definition, Vrijens et al. [16] conceptualized a framework for defining and describing medication adherence termed the Ascertaining Barriers to Compliance (ABC) taxonomy. The ABC taxonomy proposed by Vrijens et al. [16] posits that medication adherence denotes “the process by which patients take their medications as prescribed” and can be categorized into three distinct quantifiable phases: initiation, implementation, and discontinuation. The initiation phase is when the patient takes the first dose of prescribed medication. The implementation phase refers to “the extent to which a patient′s actual dosing corresponds to the prescribed dosing regimen, from initiation until the last dose.” The discontinuation phase is when the patient stops taking the prescribed medication for any reason. Persistence is the duration between initiation and discontinuation of therapy [16]. The factors influencing adherence at each phase are essential considerations when medication adherence is assessed using the ABC taxonomy [16, 17]. As indicated in the report by De Geest et al. [18] and highlighted in the review of measures by Khan and Aslani [19], “using the ABC taxonomy helps to understand the distinct phases of adherence appropriately and eliminates any ambiguity around the concept of medication adherence” [19].

The use of the ABC taxonomy is especially relevant in BC because BC is increasingly considered a chronic noncommunicable condition with multiple treatment modalities, including therapies with diverse mechanisms of action and modes of administration (e.g., OAMs). In addition, appropriate treatments or medications are recommended based on the type and stage of BC; hence, there may be a need for changes in therapy [3, 20]. Moreover, since patients or their caregivers manage OAM, problems regarding OAM adherence can arise from inconsistency in medication use [21, 22]. These problems can occur at specific phases of adherence (initiation, implementation, and discontinuation) [16].

Medication adherence is a multifaceted and dynamic process that varies over time; hence, factors influencing one adherence phase may differ from the other two. Thus, assessing the factors influencing each adherence phase is necessary to design tailored strategies to improve medication adherence [16, 23, 24]. Improving adherence to prescribed OAMs can enhance health outcomes for patients with cancer, including BC [25–27]. Evidence indicates that between 30% and 50% of patients prescribed OET are nonadherent [28], and 31%–73% of patients discontinue OET before the recommended minimum duration of 5 years [29, 30]. While optimal adherence to OET reduces the risk of BC recurrence and improves survival in women with HR+ BC, the benefits of OET are decreased in practice by nonadherence [25, 31, 32]. Nonadherence to OET increased the risk of distant metastasis, BC recurrence, worse disease‐free survival and mortality, increased medical costs, and lower quality of life years [3, 32, 33]. Nonadherence rates in patients with BC reported for oral capecitabine varied between 10% and 25% [34, 35] and may result in suboptimal outcomes, poor survival rates, potential adverse events, and increased hospitalization and cost [36]. For targeted therapy such as trastuzumab, 15% of HER2‐positive BC patients with tumors ≥ 1.0 cm were noncompliant or did not complete treatment [37]. For lapatinib, 22% of patients with metastatic BC had less than 80% of the medication (MPR < 80*%*), and nonadherence was associated with more physician visits [38]. In Africa, the reported nonadherence rates for endocrine medications varied between 4.3% and 65.4%; 80.9% for capecitabine, cyclophosphamide, and prednisolone; and 32.7% for combined medications [39].

In different contextual settings, including Africa, various factors have been reported to be associated with OAM nonadherence in patients with BC, for example, medication side effects, low income, comorbidity, depression, limited awareness of medical insurance, and mastectomy [25, 31, 39, 40]. Several reviews have shown a considerable increase in endocrine therapy adherence research in BC in the last few decades, particularly outside Africa [3, 25, 31, 40]. Although reviews of adherence to oral cytotoxic and targeted therapy in the general cancer population exist [26, 38, 41], reviews of adherence to oral cytotoxic and targeted therapy in BC are relatively scarce. In Africa, only one review has specifically evaluated adherence to oral endocrine and cytotoxic therapy in women with BC [39].

There are substantial differences in adherence assessment and measurement methods and a lack of a gold standard measure of OAM adherence in patients with cancer, including BC [3, 25, 31, 41–43]. The review of adherence to OAMs in African women with BC also reported variation in the adherence assessment methods used in the studies included [39]. The methods used to measure OAM adherence are categorized as direct and indirect or objective and subjective methods, each with its merits and demerits. Direct methods include objective measures such as measuring medication levels in plasma, serum, or urine assays using drug markers with a target medication and directly observing the patient ingesting the medication. However, direct methods are often unavailable, impractical, or too costly to use in the clinical setting [3, 25, 31, 41, 42]. Indirect methods include both objective and subjective methods. Indirect objective measures such as electronic monitoring, pill counts, pharmacy records, or prescription refills and claims data can approximate patient medication intake, but whether actual ingestion of the medication occurred is unknown, and they can be time‐consuming and often impractical in the clinical setting. Moreover, electronic monitoring systems provide relatively higher accuracy but are costly and require additional technical support. However, only patients can report reasons for taking or not taking their medications. Hence, indirect subjective measures, such as self‐reports, are commonly used to assess medication adherence, particularly in clinical practice. Self‐report measures are appealing due to their low cost, easy administration, and less time‐consuming. However, self‐reports are susceptible to social desirability, response, or recall bias [3, 25, 31, 41, 42].

Nevertheless, there is limited systematic evidence available on the methods or measures used to assess OAM adherence at specific phases in BC, as previous reviews within and outside Africa have focused on rates, factors, consequences of medication adherence or nonadherence, or interventions for improving medication adherence [3, 25, 31, 39, 40]. To our knowledge, no systematic review has reported the methods or range of measures used to assess adherence to OAMs in BC or categorized those measures according to their use at the distinct phases of adherence recommended by the ABC framework [16]. Currently, limited information is available regarding methods used to assess OAM adherence at specific phases in women with BC, particularly in Africa. Considering the complexity of measuring OAM adherence in patients with cancer, including BC [41, 42, 44], and the limited information available regarding the methods that can accurately assess OAM adherence in BC at distinct phases, it is vital to identify measures that can accurately assess OAM adherence at distinct phases and possible quality measures that could be used for future research, particularly in the African context.

This review systematically searched the literature to identify adherence measures used to assess OAM adherence in women with BC in Africa. The review sought to address the following objectives: (a) to identify the phase of adherence (initiation, implementation, or discontinuation) that has been studied most for measuring OAM adherence in women with BC and (b) to assess the methods used to measure OAM adherence at its three phases: initiation, implementation, and discontinuation.

## 2. Methods

The Joanna Briggs Institute (JBI) guidelines guided this systematic review [45]. The updated “Preferred Reporting Items for Systematic Reviews and Meta‐Analyses (PRISMA)” guidelines informed the reporting of this review [46, 47]. A systematic search was performed in English using four electronic databases: Health Source: Nursing/Academic Edition, MEDLINE (via EBSCOhost), PubMed, and Google Scholar. A literature review was systematically performed for studies that measured OAM adherence in women with BC in Africa. The database search was limited to studies in English published since 1990, as that year coincided with the OAM use, hence the emergence of adherence issues in cancer treatment [48]. The search was updated in March 2025. Six concept terms—medication adherence, oral anticancer agents, breast cancer, measures, women, and Africa—were used to search the literature. Free‐text words and MeSH terms were combined and used for each concept. Supporting Information 1 shows the detailed search strategy.

### 2.1. Eligibility Criteria

The studies reviewed met the following criteria: Studies were included if they (i) assessed OAM adherence or nonadherence rate, (ii) patients were pre‐ or postmenopausal women with BC, (iii) aged 18 years and above, (iv) sufficiently described study design and methods to allow for the categorization of the adherence phases, (v) indicated the sample in which the OAM adherence was measured, (vi) African studies reported in English and published from January 1990, and (vii) heterogeneous sample of cancer or BC patients (female and male) if findings for women with BC were presented separately. Studies were excluded if they (i) reported adherence measured in females with BC less than 18 years, men with BC, and women with cancers other than BC; (ii) the primary objective was not to measure OAM adherence; (iii) participants included heterogeneous samples of cancer patients in which findings for women with BC were not presented separately; (iv) did not report an adherence measure in the study; (v) assessed adherence to intravenous chemotherapy cycles in women with BC conducted within and outside Africa; (vi) assessed adherence to OAMs conducted outside Africa; (vii) were not published in English or were published before January 1990; and (viii) were animal, protocol, and review studies.

### 2.2. Study Selection, Data Extraction, and Quality Evaluation

The titles and abstracts of studies arising from all database searches were initially screened for potential studies based on eligibility criteria. This screening was followed by a full‐text review. Two reviewers independently evaluated each study. Any discrepancy in the eligibility of any study was resolved by a third researcher by consensus. The data was checked for consistency and validated by the other researcher [49, 50]. The same procedure was applied in the evaluation of adherence phases [16] and the methodological quality of included studies.

### 2.3. Study Selection

A two‐step process was followed to select the studies. In the first step, all duplicates were removed, and the titles and abstracts of studies identified from the search strategy were screened according to the eligibility criteria. Next, the full text of eligible studies was reviewed to confirm if they met the eligibility criteria. Eligible studies were selected according to predetermined criteria to extract relevant information. Also, the reference sections of included studies were searched for more studies that met the inclusion criteria.

### 2.4. Data Extraction and Evaluation

The relevant data from eligible studies were extracted independently by each reviewer using an adapted data extraction form. The third researcher resolved any discrepancy in data extraction through discussion and agreement [49, 50]. The information extracted from the included studies was the authors, publication year, country, study sample, study design, applied OAM adherence measure or method, criteria for adherence, and the phase of OAM adherence assessed (i.e., initiation, implementation, and discontinuation).

The phases of adherence were assessed according to the ABC conceptual framework, as proposed by Vrijens et al. [16]. The ABC framework was applied as follows: “Adherence process starts with initiation, when patients start the first dose of medication, followed by implementation, which refers to ‘the level of a patient′s actual dosing corresponding to the regimen from the first to the last dose.’ Discontinuation is regarded as when no more doses are taken thereafter” [16]. Thus, we classified studies as the “initiation phase” of adherence when participants with new prescriptions for OAMs were recruited to the study or if the study stated that participants were starting OAM treatment for the first time. Studies that evaluated OAM adherence following participants who had started OAM treatment were categorized as the “implementation phase” of adherence. Studies that assessed OAM adherence when patients stopped taking OAMs were categorized as the “discontinuation phase” of adherence [16]. The methodological quality of the reviewed studies was appraised using the Mixed Methods Appraisal Tool (MMAT) Version 2018 [51].

### 2.5. Data Synthesis

Differences in study designs and outcome measures did not support quantitative aggregation (meta‐analysis). Thus, a narrative synthesis was performed without conducting additional statistical or sensitivity analyses using specific software or without additional feasibility assessment, such as publication bias or funnel plot [3].

## 3. Results

The presentation of the results is categorized into three sections: (i) study selection, (ii) study characteristics, and (iii) results of individual studies.

### 3.1. Study Selection

**Figure 1:**
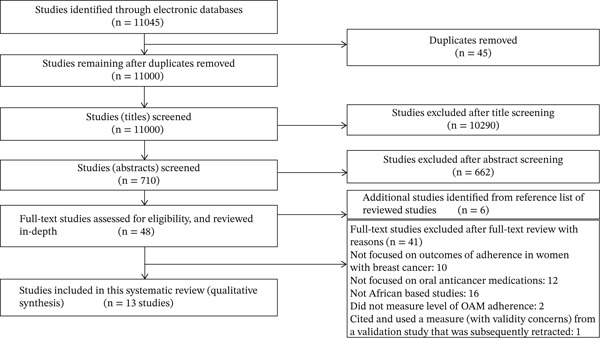
PRISMA flow diagram of study selection process.

The flow diagram of the study selection process is shown in Figure 1, based on the updated PRISMA guidelines [46, 47].

### 3.2. Study Characteristics

The general characteristics of the African studies included are presented in Table 1. The 13 studies included had a sample size of 2106 (range 26–369) patients and evaluated data on the management of nonmetastatic, recurrent, or metastatic BC. The earliest study was published in 2006 [52], and the most recent was published in 2023 [64]. Most studies collected prospective data (*n* = 9) [55–60, 62–64], and fewer collected retrospective data (*n* = 4) [52–54, 61]. Most studies (77.0%; *n* = 10) evaluated endocrine medications for BC [52–59, 62, 64], whereas two studies evaluated cytotoxic chemotherapies [60, 63], and one assessed combined endocrine and cytotoxic medications [61].

**Table 1:** Study characteristics.

Reference (country)	Study design	Medication	Study sample	Adherence measure (criteria for adherence)	Phase of adherence
[52] (Nigeria)	Retrospective (quantitative)	Tamoxifen	Female patients with breast cancer (BC) from two hospitals (*n* = 194)	Clinical records review, including physician documentation of patient report (not reported)	Implementation and discontinuation
[53] (South Africa)	Retrospective (quantitative)	Primary endocrine therapy with tamoxifen and aromatase inhibitors	Patients over 70 years of age with endocrine‐sensitive BC who refused to undergo or were not eligible for surgery (*n* = 122)	Medical records review (not reported)	Implementation
[54] (South Africa)	Retrospective (quantitative)	Endocrine therapy	Female patients newly diagnosed with BC (*n* = 250)	Medical records review (compliance: The patient was still taking endocrine therapy at the last follow‐up)	Implementation
[55] (Sudan)	Prospective (quantitative)	Tamoxifen and aromatase inhibitors	Pre‐ and postmenopausal women with nonmetastatic and metastatic BC (*n* = 172)	Patient self‐report using the Simplified Medication Adherence Questionnaire (SMAQ) and the pill count method (adherence: Taking ≥ 80% of the prescribed pills [MPR ≥ 80*%*]). Nonadherence was defined as MPR < 80*%*. Persistence: Length of time from the first prescription to a minimum break of 180 days before the completion of 5 years of therapy or the interview date	Implementation and persistence
[56] (Nigeria)	Prospective (quantitative)	Tamoxifen	Patients with BC who received tamoxifen for at least 1 year (during their first year of tamoxifen use)(*n* = 114: 108 females and six males)	Patient self‐report via questionnaire (patients were asked routine questions on nonadherence or stoppage of medication). Adherence was defined as patients not using TAM for up to 1 week without consultation with the prescribing doctors	Implementation and discontinuation
[57] (Ethiopia)	Prospective mixed methods study (quantitative and qualitative methods)The adherence measurement component was prospective (quantitative)	Tamoxifen	Women with BC who had surgery (*n* = 51) were eligible for tamoxifen therapy. *n* = 26 started tamoxifen in the adherence assessment period	Prescription refill recordsInitiation: Start of therapy. Implementation: MPR ≥ 80*%*. Discontinuation: A refill gap > 6 months. Persistence: Length of time between initiation and last dose before discontinuation	Initiation, implementation, discontinuation, and persistence
[58] (Ethiopia)	Prospective (quantitative)	Tamoxifen and anastrozole	Women with BC on adjuvant ET (*n* = 209)	Patient chart review guide and prescription refill records (paper‐based). Adherence was defined as MPR ≥ 80*%*; nonadherence: MPR < 80*%*	Implementation
[59] (Egypt)	Prospective (quantitative)	TAM and AIs (letrozole, anastrozole, and exemestane)	Pre‐ and postmenopausal female patients with endocrine receptor–positive BC (any stage) (*n* = 139)	Patient report: Interviewer‐administered questionnaire. Adherence: Number of days receiving the medications divided by the total days, expressed as a percentage. Adherence cut‐off: MPR ≥ 80*%*. Nonadherence: MPR < 80*%*. Complete adherence: MPR 90%–100%. Partial adherence: MPR 80%–< 90%	Implementation
[60] (Nigeria)	Prospective (quantitative)	Cyclophosphamide, capecitabine, and prednisolone	Women diagnosed with BC (n = 188)	Patient self‐report as documented in clinical records by the attending physician (nonadherence: Taking ≤ 90% of oral chemotherapy at any oncology clinic visit or self‐report of stoppage of medication)	Implementation and discontinuation
[61] (Ethiopia)	Retrospective (quantitative)	Capecitabine, tamoxifen, or anastrozole	Patients with a histologic diagnosis of BC who received at least one cycle of chemotherapy (*n* = 107)	Medical record review and patient report by phone interviews. Not reported: Nonadherence was classified as a drug‐related problem. No operational definition provided	Implementation
[62] (Ethiopia)	Prospective randomized controlled trial (quantitative)	Tamoxifen	Eight hospitals were randomized:Intervention group: Four hospitalsControl group (usual care): Four hospitals. Patient sample *n* = 162: (intervention group = 87; control group = 75)	Evaluation of 12 months of medication‐refill data with MPR and patient self‐report using SMAQ (Adherence: MPR ≥ 80*%*)(Discontinuation: Not having a refill in a 90‐day interval in the first 6 months or for 180 days onwards)(Persistence: Duration between initiation of therapy and last dose before discontinuation)	Implementation, discontinuation, and persistence
[63] (Kenya)	Descriptive cross‐sectional study (quantitative)	Chemotherapy, including oral chemotherapy	60 patients with Stages I–IV BC on chemotherapy	The Medication Adherence Report Scale (MARS‐5) was employed to assess chemotherapy adherence (Nonadherence was regarded as a patient′s failure to take or follow the chemotherapy medication or treatment regimen as recommended)	Implementation
[64] (South Africa)	Prospective cohort study (quantitative)	Adjuvant tamoxifen (20 mg daily dose)	369 black women with Stages I–III hormone receptor–positive breast cancer (78 were women living with HIV [WLWH] and 291 were HIV‐negative)	Analysis of concentrations of tamoxifen and its metabolites in venous blood samples using a triple quadrupole mass spectrometer (Nonadherence was defined as a tamoxifen level < 60 ng/mL after 3 months of daily tamoxifen use)	Implementation and persistence

Tables 2 and 3 outline the measures and phases of OAM adherence (initiation, implementation, and discontinuation) and persistence in women with BC.

**Table 2:** Methods used to measure OAM adherence at its three phases and the availability of reliability and validity data of the measures.

Reference (country)	Adherence assessment methods						Reliability			Validity			Adherence phase(s) assessed			
	Objective methods				Subjective methods		Provided reliability data	Referred to other	No data	Provided validity data	Referred to other	No data	Initiation	Implementation	Discontinuation	Persistence
	Indirect			Direct	Patient report											
	Pharmacy record (prescription refill)	Pill count	Medical record review	Drug assays in blood	Standardized self‐report questionnaire	Nonstandardized items										
						Self‐report items designed by the study team										
[57] (Ethiopia)	X												X	X	X	X
[62] (Ethiopia)	X				X (SMAQ)			Yes			Yes			X	X	X
[58] (Ethiopia)	X													X		
[55] (Sudan)		X			X (SMAQ)									X		X
[59] (Egypt)						X								X		
[60] (Nigeria)						X								X		
[56] (Nigeria)						X								X	X	
[54] (South Africa)			X											X		
[53] (South Africa)			X											X		
[61] (Ethiopia)			X											X		
[52] (Nigeria)			X											X	X	
[63] (Kenya)					X (MARS‐5)									X		
[64] (South Africa)				X (concentrations of tamoxifen and its metabolites in venous blood)										X		X

*Note:* This study [62] used SMAQ to measure adherence but did not provide validity or reliability data for the patient sample assessed in Ethiopia. However, the study [62] referred to another study by Oberguggenberger et al. [65] that had evaluated SMAQ for endocrine therapy adherence for a patient sample outside Africa and reported a Cronbach′s alpha of 0.67 for reliability but did not report validity data. This study [55] used SMAQ to measure OAM adherence but did not report data on reliability or validity for the patient sample assessed in Sudan. This study [63] used MARS‐5 to measure chemotherapy adherence but did not provide the reliability and validity data for the patient sample assessed in Kenya.

Abbreviations: AHT, adjuvant hormonal therapy; MARS‐5, Medication Adherence Report Scale; SMAQ, Simplified Medication Adherence Questionnaire.

**Table 3:** Measures used to assess OAM adherence in its three phases.

Adherence phase	Adherence measures	Total
Initiation	Pharmacy record using prescription refill data [57]	1

Implementation	Pharmacy record using prescription refill data [57, 58, 62]	3
	Pill count [55]	1
	Medical record review [52–54, 61]	4
	Self‐report questionnaire [55, 56, 59, 60, 62, 63]	6
	Drug assay in blood [64]	1

Discontinuation	Pharmacy record using prescription refill data [57, 62]	2
	Medical record review [52]	1
	Self‐report [56, 62]	2

Persistence	Permitted gap period (grace period)	
	180 days [55]	1
	No permitted gap period was reported [57, 62, 64]	3

Of the 13 studies included, seven used only objective measures [52–54, 57, 58, 61, 64], four used only subjective measures [56, 59, 60, 63], and two combined objective and subjective measures [55, 62] to assess OAM adherence. All the studies reviewed assessed OAM adherence at the implementation phase, followed by the discontinuation phase (30.8%, *n* = 4) [52, 56, 57, 62] and the initiation phase [57]. Nine studies measured one phase of adherence [53–55, 58–61, 63, 64]. Three studies measured two phases of adherence [52, 56, 62], and one study measured three phases [57]. Additionally, persistence was reported in four studies (30.8%, *n* = 4) [55, 57, 62, 64]. The appraisal of the studies indicates that overall, most studies (nine) demonstrated high [57, 58, 62–64] to moderate [55, 56, 59, 61] quality, and four were of low quality [52–54, 60] (Supporting Information 2).

### 3.3. Results of Individual Studies

The results of individual studies are summarized in Table 1. Tables 2 and 3 outline the reported adherence measures used in assessing the initiation, implementation, and discontinuation phases of OAM adherence and persistence in women with BC.

### 3.4. Methods Used to Measure Adherence at the Different Phases

#### 3.4.1. Measures Used at the Initiation Phase

Only one study assessed adherence at the initiation phase [57]. Medication initiation was defined as the date when the first prescription of tamoxifen was dispensed (i.e., the date of the first handover of tamoxifen was regarded as the start of therapy). However, there was no information to indicate that the patients had started tamoxifen on the date their prescription was dispensed. The study collected prospective data and obtained information from pharmacy records (prescription refills). The study did not report a cut‐off period for patients to start the medication, yet patients were considered adherent if they started the medication within the adherence assessment period.

#### 3.4.2. Measures Used at the Implementation Phase

All 13 studies identified several adherence measures during the implementation phase. Subjective measures such as self‐reports were the most commonly used (46.2%; *n* = 6) [55, 56, 59, 60, 62, 63]. Standardized instruments such as the Simplified Medication Adherence Questionnaire (SMAQ) were used by two studies [55, 62], the Medication Adherence Report Scale (MARS‐5) was used by one study [63], and three studies used nonstandardized self‐report items designed by the study team [56, 59, 60]. Different objective measures (69.2%; *n* = 9) were used to assess OAM adherence at the implementation phase and included prescription refill (21.4%; *n* = 3) [57, 58, 62], pill count (7.7%; *n* = 1) [55], medical records review (30.8%; *n* = 4) [52–54, 61], and drug assays in blood (7.7%; *n* = 1) [64]. However, two studies (15.4%) used multiple measures (objective and subjective), including pill count and SMAQ [55], and prescription refill and SMAQ [62], respectively, to assess OAM adherence at the implementation phase.

#### 3.4.3. Measures Used at the Discontinuation Phase

Adherence to OAM at the discontinuation phase was measured in four studies [52, 56, 57, 62]. Reibold et al. [57] used prescription refills to measure tamoxifen discontinuation, while Getachew et al. [62] combined prescription refills with SMAQ to measure tamoxifen discontinuation. The two studies used different cut‐off periods to determine discontinuation. Reibold et al. [57] considered more than 6‐month intervals (refill gap > 6 months) to indicate tamoxifen discontinuation. Getachew et al. [62] used two cut‐offs to determine discontinuation. They considered tamoxifen discontinuation if the prescription was not refilled within a 90‐day interval in the first 6 months or for 180 days onwards. Oguntola et al. [56] used nonstandardized self‐report items to measure the discontinuation of tamoxifen in the first 6 months of treatment commencement. Adesunkanmi et al. [52] reviewed medical records, including physician documentation of patient reports in medical records, to assess tamoxifen discontinuation.

#### 3.4.4. Measures Used in Assessing Persistence

Persistence was measured in four studies [55, 57, 62, 64], and methods for calculating persistence varied across studies (e.g., whether a permissible gap period was reported or not reported). Two studies defined persistence in continuous form (months persistent or nonpersistent with medication) [55, 64]. One study reported a permissible gap in medication therapy [55], whereas the other did not [64]. Two studies defined persistence in a discrete form (proportion of patients persistent or nonpersistent with the medication) with no reported permissible gap period in medication therapy [57, 62]. Mohamed and Elamin [55] used pill count and SMAQ to measure persistence and calculated persistence as the length of time from the first prescription to a break of at least 180 days before completing 5 years of therapy or the interview date. Their study applied a permissible gap in medication therapy of at least 180 days. Ayeni et al. [64] used drug assays in blood to measure concentrations of tamoxifen and its metabolites and calculated persistence as the length of time in months since tamoxifen initiation (median [interquartile range] time of 13.0 [6.2–25.2] months), with no reported permissible gap in medication therapy. Reibold et al. [57] used prescription refills and defined persistence as the length of time between initiation and last dose before discontinuation, with no reported permissible gap in medication therapy. Getachew et al. [62] used prescription refills and SMAQ. They defined persistence as the duration between initiating therapy and the last dose before discontinuation, with no reported permissible gap in medication therapy.

#### 3.4.5. Psychometric Properties (Reliability and Validity) of Adherence Measures

Table 2 shows that among the 13 included studies, one study from Kenya [63] that used the standardized MARS‐5 stated that reliability and validity were assessed but did not report the reliability and validity data for the BC patient sample assessed. Furthermore, in 12 studies, researchers did not report data on the reliability and validity of adherence measures for the BC patient sample in Africa. Although one study from Sudan [55] used the standardized SMAQ to measure adherence to tamoxifen or AIs, the study did not report validity or reliability data of the SMAQ for the patient sample assessed. Another study from Ethiopia [62] that also used standardized SMAQ to measure tamoxifen adherence did not provide reliability or validity data for the BC patient sample evaluated. Instead, the Ethiopian study [62] cited a previous study [65] that evaluated SMAQ for endocrine therapy adherence for a BC patient sample outside Africa that reported reliability data (Cronbach′s alpha of 0.67) but not validity data.

## 4. Discussion

This review comprehensively examined the methods used to measure OAM adherence in women with BC. The measures were mapped to the specific phases of adherence according to the ABC framework: initiation, implementation, and discontinuation [16]. This review has thus summarized and bridged the gap in the OAM adherence literature by identifying the measurement methods based on their use at the specific phases of adherence. Adherence to OAM was mainly measured at the implementation phase, with few studies assessing OAM adherence at the initiation and discontinuation phases. Moreover, few studies evaluated persistence with OAMs. Of all the studies included, only one study used a direct objective method (drug assays in blood) and the other studies used indirect (objective or subjective) methods to measure OAM adherence. Self‐report, an indirect subjective method, was the most common assessment method. However, different assessment methods seem to capture various facets of medication‐taking behavior. Thus, multiple measures are recommended that integrate subjective (e.g., validated self‐report questionnaire) and objective (e.g., prescription refill) approaches. Direct methods, such as medication levels in plasma or drug assays of serum or urine, the use of drug markers with a target medication, and directly observing the patient ingesting the medication, though costly and sometimes impractical in clinical settings, can be used when accurate measurement of adherence is essential for assessment of outcome, as direct measures can capture information missed by indirect measures [31, 42]. Self‐report adherence measures should be triangulated with objective methods, as self‐reports have been shown to overestimate adherence to OAMs [25, 31].

Prescription refill was used in one study that reported adherence at the initiation phase [52] to assess when the first prescription of the OAM was dispensed. However, the study did not report whether the patient started OAM on the date the prescription was dispensed, nor was a cut‐off period used to determine initiation. Patients were only considered adherent if they started the medication within the adherence assessment period, suggesting that there was no information on whether there was a delay in initiating OAM in women with BC. However, the actual period when patients initiate OAM should be reported in studies to determine if there are delays in starting prescribed OAM and the possible reasons for such delays. The information can help design strategies to encourage early OAM initiation and minimize delay in treatment. The information on the OAM initiation period may help identify the outcomes of early initiation, delayed initiation, or noninitiation of OAM. Evidence suggests that a shorter time to treatment initiation (TTI) was associated with lower mortality for patients with common cancers, including BC [66]. Moreover, treatment delay was associated with increased mortality in patients with cancer, including BC [67]. Hence, future research should focus on assessing OAM initiation to identify early initiation, delayed initiation, or noninitiation; understanding the reasons for the behavior; and the effects on patient outcomes. The information could guide strategies to improve initiation and outcomes in women with BC.

While prescription refill records offer a vital way to measure initiation, directly asking women with BC when they started the prescribed OAM provides a direct means of measuring initiation (and triangulating quantitative refill records) and obtaining a comprehensive understanding of the factors influencing OAM initiation. One limitation of refill records is the assumption that the patient would consume OAM after the refill. However, the information in the records does not indicate the actual medication‐taking behavior of the patients [42]. Knowledge of when the patient took the OAM is vital in the initiation phase because it assists the clinician in assessing the medication′s effectiveness. Self‐report measures have the advantages of low cost, minimal technical obstacles, obtaining immediate feedback from the patient, and being appropriate in real‐life clinical settings. They remain a potential opportunity for future assessment of OAM initiation. Moreover, developing a multimeasure approach (combining objective and subjective measures) is another way to increase our understanding of the initiation and the reasons for delayed or noninitiation of medication [68]. This understanding can assist in designing strategies to support OAM initiation. Nevertheless, further research is needed in the context of OAM adherence in BC to determine the validity and reliability of applying multiple measures at the initiation phase of therapy.

Implementation was the most assessed phase of OAM adherence in women with BC. Implementation is a continuous process that requires long‐term assessment over time and can be used to quantify adherence or nonadherence and determine the types of nonadherence [19, 69, 70]. Different methods were used to measure implementation, and patient self‐report was the most commonly used method. Similar findings of using self‐report were reported by other reviews outside Africa that measured medication adherence in different disease conditions, such as in patients with attention‐deficit hyperactivity disorder (ADHD) [19] or in patients with unipolar depression [71]. In contrast, a systematic review of adherence to cancer treatment in older adults showed that over half (56%) of the included studies used data from several administrative and clinical databases or chart reviews (using claim codes and prescription refill data) [72]. The self‐report, SMAQ, was used in two studies assessing OAM adherence in women with BC in Africa [55, 62]. However, SMAQ was not explicitly designed for BC but was initially developed to assess medication‐taking behavior and barriers among HIV‐infected adult patients [73–75]. Other self‐report items used to determine OAM adherence in BC in the included African studies were nonstandardized items [56, 59, 60].

Although implementation was the most commonly assessed adherence phase, differences in the reported levels of nonadherence were observed among studies with the same or different measurement methods, as shown in the previous review from Africa [39]. The differences in adherence measurement could be due to subjective and objective methods. Subjective methods, such as self‐reports, have been observed to typically overestimate adherence due to social desirability, response, or recall bias compared to objective methods. With self‐report measures, differences in results could be explained by the distinct functions of questionnaires and scales [69, 70]. Some self‐report measures, including questionnaires, are more disease‐ or context‐specific and may not be appropriate, valid, or reliable in other disease conditions or contexts [69, 70]. Self‐report measures are low‐cost, easy to administer, noninvasive, flexible in the mode of administration, have minimal patient burden, and are less time‐consuming for assessing the implementation of a medication regimen over time. However, they are influenced by social desirability, response, or recall bias. Studies can minimize social desirability bias by introducing the measure with a statement that normalizes nonadherence and encourages honest responses, designing study questions that enable recall and are valid and reliable [69, 70, 73].

Different methods were used to measure OAM adherence at the discontinuation phase. The operational definitions of OAM discontinuation and nonpersistence in BC research differ and partly overlap, contributing to methodological limitations in OAM adherence research in BC treatment. Persistence to OAM and discontinuation in women with BC were measured in the included studies using prescription refill records, pill count, drug assays in blood, or self‐report (SMAQ). However, as data sources, these approaches have some limitations. The prescription refill method [57, 62], self‐report method [62], and the use of drug assays in the blood [64] applied in these studies fail to differentiate discontinuation from nonpersistence due to the failure to report using a permissible gap period [57, 62]. However, the study from Sudan [55] that used the pill count and self‐report (SMAQ) method did not report discontinuation but reported nonpersistence and applied a permissible gap period in medication therapy of 180 days.

The few studies that evaluated OAM persistence defined persistence in terms of continuing medication from initiation to discontinuation. Still, a clear definition of initiation (start of dosing) and discontinuation (an endpoint) was not often presented. Since persistence is a time‐to‐event variable, it is essential to clearly define both initiation and discontinuation to enhance the accuracy of persistence measurement. The use of a permissible gap (length of time) between prescriptions or no permissible gap in some studies is an area that demands consideration. The use of a permissible gap period of at least 180 days was reported in one study [55], whereas in other studies, no permitted gap period was reported. This difference in how persistence is measured can contribute to inconsistency in persistence measurement and difficulty in comparing studies of OAM adherence in BC.

Moreover, due to using a permissible gap period or no reported permissible gap period in some studies, a patient regarded as persistent to OAM in one study may not fulfill the criteria in another study. Furthermore, the consideration of drug holidays (planned temporary suspension of OAMs) is also missing in studies using prescription refills, pill count, and self‐report measures. Drug holidays may be an essential aspect of BC treatment as patients on cycled oral chemotherapy, such as capecitabine, do not take medication during a break in their medication cycle (rest period). Not paying attention to this facet of OAM treatment may lead to overestimating discontinuation and nonpersistence. Self‐report measures can help overcome these challenges because they can provide an opportunity to directly ask patients whether they are on drug holidays or have stopped taking OAM completely. Moreover, a self‐report measure can offer valuable insight into the reasons for discontinuing or being nonpersistent with OAMs.

Adherence assessment based on the specific phases can allow clinicians to recognize and tackle the phase‐specific factors that influence OAM adherence. For instance, evaluation of adherence at the initiation phase could assist clinicians in knowing whether the patient has started the OAM and the exact date of initiation. Some patients may commence treatment immediately after receiving the prescription, whereas others may delay treatment initiation for days, weeks, or months for different reasons. The knowledge of the precise therapy date, including medication initiation, can assist clinicians in adequately assessing therapy and minimizing unnecessary treatment changes [66, 67, 76]. Identifying factors at each adherence phase can assist in designing appropriately tailored interventions that are most suitable for the factors influencing adherence at the given phase. For instance, if high medication cost is an issue for medication initiation or prevents patients from using their medication as prescribed or from continuing with their OAMs (e.g., adjuvant endocrine therapy), then interventions comprising policy changes involving lowering of medication costs can promote adherence, as reported in a systematic review [25].

For studies that used self‐reports (questionnaires) [55, 56, 59, 60, 62, 63] to measure OAM adherence in BC patients in Africa, most did not report the psychometric properties of the questionnaires used. This finding is similar to previous reviews in other disease conditions conducted outside Africa, such as the review of adherence measures in patients with ADHD, where the psychometric properties were not reported in about 50% of studies included that used self‐report measures [19], and the review of adherence measures in patients with unipolar depression, where no standout measure with strong psychometric properties was found [71].

No gold standard measure of adherence exists, nor is there a measure with good quality indicators that captures adherence across the three phases. Therefore, a multimeasure approach, including subjective and objective measures, is recommended as a practical approach to assessing adherence in patients with BC, similar to suggestions from other studies [19, 69–71]. Nevertheless, when clinicians or researchers choose to use a single measure, including self‐report, they should consider the validity, reliability, practicality, or cost‐effectiveness before selecting an adherence measure, as suggested by other studies [19, 69, 70]. Moreover, self‐report measures are regarded as the most practical method to assess adherence. Their continued use is evidence of their usefulness in clinical research, as observed in other studies [19, 69, 70]. Therefore, self‐report measures with optimal psychometric properties should be used to measure OAM adherence in BC in clinical and research settings to ensure the findings′ validity and reliability. Researchers are encouraged to report the validity and reliability of adherence measures, including questionnaires used in their studies, thus highlighting the need to standardize adherence measurement to enhance data comparability across studies, develop tailored interventions to improve adherence, and optimize patient outcomes.

The lack of reliable and well‐validated methods, including standardized questionnaires and scales designed to assess or measure OAM adherence in women with BC in Africa, may contribute to the conceptual and methodological limitations observed in OAM adherence research in BC patients. Adherence questionnaires can potentially explore medication‐taking behaviors, provided the right questionnaire or scale is selected or designed based on the study objectives, target population, adherence phase, and psychometric properties, as reported in previous studies [19, 69, 70].

The limitation of most of the existing self‐report measures, including questionnaires used in the included studies, is that they confound two related but different nonadherence constructs: the extent to which doses are missed (adherence behavior) and the reasons for missing doses [77–79]. Each construct is assessed by a different type of psychometric model, with essential measurement implications, in the sense that the psychometric model by which each construct is assessed dictates how to establish reliability and validity. However, most existing self‐report measures were not developed with this distinction, compromising validity and reliability [77–79]. Given the inadequacies in the available questionnaires, there is a need to develop a disease‐ and context‐specific measure that is efficient and able to measure adherence across the three specific phases and detect changes in medication‐taking behavior over time in patients with BC in Africa.

## 5. Strengths and Limitations

This review examined the methods used to measure OAM adherence in its three phases based on the ABC taxonomy. We applied a comprehensive search strategy with clear eligibility criteria, a transparent approach to data collection, and a two‐step review of the included studies according to the eligibility criteria. The reporting followed the updated PRISMA guidelines [46, 47], contributing to the robustness of our findings. However, this review has some limitations. Differences in study designs, measurement methods, and criteria for adherence did not support quantitative synthesis (meta‐analysis) of the results of included studies. Some potentially relevant studies might have been omitted, as non‐English studies were not included. The review protocol was not formally registered due to the commencement of the systematic literature search before protocol registration and time constraints. Nevertheless, our findings provide relevant insights into the specific phases of adherence and the methods used to measure OAM adherence in women with BC. Thus, this systematic review potentially contributes to the future standardization of OAM adherence research in women with BC in Africa.

## 6. Conclusion

Implementation was the most common phase of adherence assessed, while initiation and discontinuation were relatively less assessed. Self‐report measures, including questionnaires, were the most common OAM adherence assessment method. However, for most reviewed studies, the psychometric properties, including reliability and validity of the self‐report measures, were not reported for women with BC in Africa. The study findings can inform and guide clinicians and researchers in selecting or developing appropriate measures with sound psychometric properties for assessing OAM adherence based on the specific phases of adherence. Therefore, this review suggests the need to develop more valid and reliable adherence measures, including self‐report questionnaires, that can potentially assess OAM adherence across its three phases, particularly in the African context. Generating phase‐specific data can guide the design of tailored strategies to enhance OAM adherence in women with BC. When phase‐specific strategies are designed based on identified relevant factors, they are more likely to optimize OAM adherence and patient outcomes effectively.

## Author Contributions

D.O.O., M.J.C., and E.B.O. contributed to the conception and design of the study. D.O.O. performed background data collection, analysis, and interpretation. T.M., M.J.C., and E.B.O. contributed to data analysis and interpretation. E.B.O. and M.J.C. provided supervision. D.O.O. wrote the initial draft of the manuscript.

## Funding

This study was funded by the College of Health Sciences, University of KwaZulu‐Natal (10.13039/501100024216) (636726).

## Disclosure

The protocol for this systematic review was not registered or published elsewhere. All authors reviewed the preceding versions of the manuscript. All authors approved the final manuscript.

## Conflicts of Interest

The authors declare no conflicts of interest.

## Supporting Information

Additional supporting information can be found online in the Supporting Information section.

## Supporting information


**Supporting Information 1** Search strategy.

**Supporting Information 2** Quality appraisal.

## Data Availability

The article and supporting information contain the data and other materials supporting this study.
