# Supplementary material for: A Systematic Review of Methods to Measure Adherence to Oral Anticancer Medications in African Women With Breast Cancer at Initiation, Implementation, and Discontinuation of Therapy
Source: Int J Breast Cancer. 2026 Feb 27;2026:5293415. doi: 10.1155/ijbc/5293415 (PMC12948728; doi:10.1155/ijbc/5293415)
Supplement: Supplementary file 1 — Supporting Information 1 Search strategy. [file IJBC-2026-5293415-s001.docx]

**A Systematic Review of Methods to Measure Adherence to Oral Anticancer Medications in Women with Breast Cancer at Initiation, Implementation, and Discontinuation of Therapy**

Deborah O. Onwusah,^1^ Tafadzwa Mindu,^2^ Moses J. Chimbari,^2,3^ Elizabeth O. Ojewole^1^

**Appendix 1 - Search strategy for the Databases: 9 MARCH 2025**

**PubMed Search**

Search Results: 8,360 Time:11:43:07

((((("Medication adherence" OR "Patient compliance" OR "Adherence, Medication" OR "Medication Nonadherence" OR "Nonadherence, Medication" OR "Medication Noncompliance" OR "Noncompliance, Medication" OR "Medication Non-Adherence" OR "Medication Non Adherence" OR "Non-Adherence, Medication" OR "Medication Persistence" OR "Persistence, Medication" OR "Medication Compliance" OR "Compliance, Medication" OR Adhere* OR Medication adhere* OR Medication complian* OR Patient complian* OR Patient adhere* OR Drug adhere*) AND ("Administration, oral" OR "Drug Administration, Oral" OR "Oral Drug Administration" OR "Oral Administration" OR "Administrations, Oral" OR "Oral Administrations" OR "Administration, Oral Drug" OR "Administrations, Oral Drug" OR "Drug Administrations, Oral" OR "Oral Drug Administrations" OR Oral* OR Tablet* OR Capsule* OR Pill* OR po OR Mouth OR Buccal OR "Antineoplastic agents" OR "Agents, Antineoplastic" OR "Antineoplastic Drugs" OR "Drugs, Antineoplastic" OR "Antineoplastics" OR "Chemotherapeutic Anticancer Drug" OR "Drug, Chemotherapeutic Anticancer" OR "Antitumor Drugs" OR "Drugs, Antitumor" OR "Cancer Chemotherapy Agents" OR "Agents, Cancer Chemotherapy" OR "Chemotherapy Agents, Cancer" OR "Cancer Chemotherapy Drugs" OR "Chemotherapy Drugs, Cancer" OR "Drugs, Cancer Chemotherapy" OR "Chemotherapeutic Anticancer Agents" OR "Agents, Chemotherapeutic Anticancer" OR "Anticancer Agents" OR "Agents, Anticancer" OR "Antitumor Agents" OR "Agents, Antitumor" OR "Antineoplastic agents, Hormonal" OR "Aromatase inhibitors" OR Tamoxifen OR "Chemotherapy, Adjuvant" OR Capecitabine OR "Inhibitors, Aromatase" OR Endocrine therapy* OR Hormonal therapy* OR Adjuvant Hormonal therapy* OR Non-endocrine therapy* OR Oral chemotherapy* OR Adjuvant Chemotherapy* OR Adjuvant Drug therapy* OR Chemotherapy* OR Anticancer Medication* OR Anticancer Therapy* OR Anticancer Drug treatment*)) AND ("Breast Neoplasms" OR "Breast Neoplasm" OR ("Neoplasm, Breast")) OR ("Breast Tumors" OR "Breast Tumor" OR "Tumor, Breast" OR "Tumors, Breast" OR "Neoplasms, Breast" OR "Breast Cancer" OR "Cancer, Breast" OR "Mammary Cancer" OR "Cancer, Mammary" OR "Cancers, Mammary" OR "Mammary Cancers" OR "Malignant Neoplasm of Breast" OR "Breast Malignant Neoplasm" OR "Breast Malignant Neoplasms" OR "Malignant Tumor of Breast" OR "Breast Malignant Tumor" OR "Breast Malignant Tumors" OR "Cancer of Breast" OR "Cancer of the Breast" OR "Mammary Carcinoma, Human" OR "Carcinoma, Human Mammary" OR "Carcinomas, Human Mammary" OR "Mammary Carcinomas, Human" OR "Human Mammary Carcinoma" OR "Mammary Neoplasms, Human" OR "Human Mammary Neoplasm" OR "Human Mammary Neoplasms" OR "Neoplasm, Human Mammary" OR "Neoplasms, Human Mammary" OR "Mammary Neoplasm, Human" OR "Human Mammary Carcinomas" OR "Breast Carcinoma" OR "Breast Carcinomas" OR "Carcinoma, Breast" OR "Carcinomas, Breast")) AND (Measure* OR Assess* OR Evaluat* OR Method* OR Instrument* OR Scale* OR Survey OR Tools OR Questionnaire*)) AND (Women OR Female OR Woman OR "Women Groups" OR Women Groups OR "Women's Group" OR Women's Group OR Girls OR Girl)) AND (Africa OR Africa* OR "Developing countries" OR Saharan* OR Algeria OR Angola OR Benin OR Botswana OR "Burkina Faso" OR Burundi OR "Cabo Verde" OR Cameroon OR "Central African Republic" OR Chad OR Comoros OR "Democratic Republic of the Congo" OR "Republic of the Congo" OR "Cote d'Ivoire" OR Djibouti OR Egypt OR "Equatorial Guinea" OR Eritrea OR Eswatini OR Swaziland OR Ethiopia OR Gabon OR Gambia OR Ghana OR Guinea OR "Guinea-Bissau" OR Kenya OR Lesotho OR Liberia OR Libya OR Madagascar OR Malawi OR Mali OR Mauritania OR Mauritius OR Morocco OR Mozambique OR Namibia OR Niger OR Nigeria OR Rwanda OR "Sao Tome and Principe" OR Senegal OR Seychelles OR "Sierra Leone" OR Somalia OR "South Africa" OR "South Sudan" OR Sudan OR Tanzania OR Togo OR Tunisia OR Uganda OR Zambia OR Zimbabwe) Filters: English, Humans, Female, from 1990/1/1 - 2025/3/9

(((("Medication adherence"[All Fields] OR "Patient compliance"[All Fields] OR "adherence medication"[All Fields] OR "Medication Nonadherence"[All Fields] OR "nonadherence medication"[All Fields] OR "Medication Noncompliance"[All Fields] OR "noncompliance medication"[All Fields] OR "medication non adherence"[All Fields] OR "medication non adherence"[All Fields] OR "non adherence medication"[All Fields] OR "Medication Persistence"[All Fields] OR "persistence medication"[All Fields] OR "Medication Compliance"[All Fields] OR "compliance medication"[All Fields] OR "adhere*"[All Fields] OR (("medic"[All Fields] OR "medical"[All Fields] OR "medicalization"[MeSH Terms] OR "medicalization"[All Fields] OR "medicalizations"[All Fields] OR "medicalize"[All Fields] OR "medicalized"[All Fields] OR "medicalizes"[All Fields] OR "medicalizing"[All Fields] OR "medically"[All Fields] OR "medicals"[All Fields] OR "medicated"[All Fields] OR "medication s"[All Fields] OR "medics"[All Fields] OR "pharmaceutical preparations"[MeSH Terms] OR ("pharmaceutical"[All Fields] AND "preparations"[All Fields]) OR "pharmaceutical preparations"[All Fields] OR "medication"[All Fields] OR "medications"[All Fields]) AND "adhere*"[All Fields]) OR (("medic"[All Fields] OR "medical"[All Fields] OR "medicalization"[MeSH Terms] OR "medicalization"[All Fields] OR "medicalizations"[All Fields] OR "medicalize"[All Fields] OR "medicalized"[All Fields] OR "medicalizes"[All Fields] OR "medicalizing"[All Fields] OR "medically"[All Fields] OR "medicals"[All Fields] OR "medicated"[All Fields] OR "medication s"[All Fields] OR "medics"[All Fields] OR "pharmaceutical preparations"[MeSH Terms] OR ("pharmaceutical"[All Fields] AND "preparations"[All Fields]) OR "pharmaceutical preparations"[All Fields] OR "medication"[All Fields] OR "medications"[All Fields]) AND "complian*"[All Fields]) OR (("patient s"[All Fields] OR "patients"[MeSH Terms] OR "patients"[All Fields] OR "patient"[All Fields] OR "patients s"[All Fields]) AND "complian*"[All Fields]) OR (("patient s"[All Fields] OR "patients"[MeSH Terms] OR "patients"[All Fields] OR "patient"[All Fields] OR "patients s"[All Fields]) AND "adhere*"[All Fields]) OR ("Drug"[All Fields] AND "adhere*"[All Fields])) AND ("administration oral"[All Fields] OR "drug administration oral"[All Fields] OR "Oral Drug Administration"[All Fields] OR "Oral Administration"[All Fields] OR "administrations oral"[All Fields] OR "Oral Administrations"[All Fields] OR "administration oral drug"[All Fields] OR ("administration, oral"[MeSH Terms] OR ("administration"[All Fields] AND "oral"[All Fields]) OR "Oral Administration"[All Fields] OR ("administrations"[All Fields] AND "oral"[All Fields] AND "Drug"[All Fields])) OR ("administration, oral"[MeSH Terms] OR ("administration"[All Fields] AND "oral"[All Fields]) OR "Oral Administration"[All Fields] OR ("Drug"[All Fields] AND "administrations"[All Fields] AND "oral"[All Fields])) OR "Oral Drug Administrations"[All Fields] OR "oral*"[All Fields] OR "tablet*"[All Fields] OR "capsule*"[All Fields] OR "pill*"[All Fields] OR ("poisoning"[MeSH Subheading] OR "poisoning"[All Fields] OR "po"[All Fields]) OR ("mouth"[MeSH Terms] OR "mouth"[All Fields] OR "mouths"[All Fields] OR "mouth s"[All Fields] OR "mouthed"[All Fields] OR "mouthful"[All Fields] OR "mouthfuls"[All Fields] OR "mouthing"[All Fields]) OR ("buccal"[All Fields] OR "buccally"[All Fields]) OR "Antineoplastic agents"[All Fields] OR "agents antineoplastic"[All Fields] OR "Antineoplastic Drugs"[All Fields] OR "drugs antineoplastic"[All Fields] OR "Antineoplastics"[All Fields] OR "Chemotherapeutic Anticancer Drug"[All Fields] OR ("Antineoplastic agents"[Pharmacological Action] OR "Antineoplastic agents"[MeSH Terms] OR ("antineoplastic"[All Fields] AND "agents"[All Fields]) OR "Antineoplastic agents"[All Fields] OR ("Drug"[All Fields] AND "chemotherapeutic"[All Fields] AND "anticancer"[All Fields])) OR "Antitumor Drugs"[All Fields] OR "drugs antitumor"[All Fields] OR "Cancer Chemotherapy Agents"[All Fields] OR ("Antineoplastic agents"[Pharmacological Action] OR "Antineoplastic agents"[MeSH Terms] OR ("antineoplastic"[All Fields] AND "agents"[All Fields]) OR "Antineoplastic agents"[All Fields] OR ("agents"[All Fields] AND "cancer"[All Fields] AND "chemotherapy"[All Fields])) OR ("Antineoplastic agents"[Pharmacological Action] OR "Antineoplastic agents"[MeSH Terms] OR ("antineoplastic"[All Fields] AND "agents"[All Fields]) OR "Antineoplastic agents"[All Fields] OR ("chemotherapy"[All Fields] AND "agents"[All Fields] AND "cancer"[All Fields])) OR "Cancer Chemotherapy Drugs"[All Fields] OR "chemotherapy drugs cancer"[All Fields] OR "drugs cancer chemotherapy"[All Fields] OR "Chemotherapeutic Anticancer Agents"[All Fields] OR ("Antineoplastic agents"[Pharmacological Action] OR "Antineoplastic agents"[MeSH Terms] OR ("antineoplastic"[All Fields] AND "agents"[All Fields]) OR "Antineoplastic agents"[All Fields] OR ("agents"[All Fields] AND "chemotherapeutic"[All Fields] AND "anticancer"[All Fields])) OR "Anticancer Agents"[All Fields] OR "agents anticancer"[All Fields] OR "Antitumor Agents"[All Fields] OR "agents antitumor"[All Fields] OR "antineoplastic agents hormonal"[All Fields] OR "Aromatase inhibitors"[All Fields] OR ("tamoxifen"[MeSH Terms] OR "tamoxifen"[All Fields] OR "tamoxifene"[All Fields] OR "tamoxifen s"[All Fields] OR "tamoxifens"[All Fields]) OR "chemotherapy adjuvant"[All Fields] OR ("capecitabine"[MeSH Terms] OR "capecitabine"[All Fields] OR "capecitabin"[All Fields]) OR "inhibitors aromatase"[All Fields] OR (("endocrinal"[All Fields] OR "endocrine system"[MeSH Terms] OR ("endocrine"[All Fields] AND "system"[All Fields]) OR "endocrine system"[All Fields] OR "endocrine"[All Fields] OR "endocrines"[All Fields] OR "endocrinic"[All Fields] OR "endocrinous"[All Fields]) AND "therapy*"[All Fields]) OR (("hormon"[All Fields] OR "hormonal"[All Fields] OR "hormonally"[All Fields] OR "hormonals"[All Fields] OR "hormone s"[All Fields] OR "hormones"[Pharmacological Action] OR "hormones"[MeSH Terms] OR "hormones"[All Fields] OR "hormone"[All Fields] OR "hormons"[All Fields]) AND "therapy*"[All Fields]) OR (("adjuvancy"[All Fields] OR "adjuvanted"[All Fields] OR "adjuvanting"[All Fields] OR "adjuvants"[All Fields] OR "adjuvants, pharmaceutic"[MeSH Terms] OR ("adjuvants"[All Fields] AND "pharmaceutic"[All Fields]) OR "pharmaceutic adjuvants"[All Fields] OR "adjuvant"[All Fields] OR "adjuvants, immunologic"[MeSH Terms] OR ("adjuvants"[All Fields] AND "immunologic"[All Fields]) OR "immunologic adjuvants"[All Fields] OR "adjuvated"[All Fields] OR "adjuvation"[All Fields] OR "adjuvent"[All Fields]) AND ("hormon"[All Fields] OR "hormonal"[All Fields] OR "hormonally"[All Fields] OR "hormonals"[All Fields] OR "hormone s"[All Fields] OR "hormones"[Pharmacological Action] OR "hormones"[MeSH Terms] OR "hormones"[All Fields] OR "hormone"[All Fields] OR "hormons"[All Fields]) AND "therapy*"[All Fields]) OR ("Non-endocrine"[All Fields] AND "therapy*"[All Fields]) OR (("mouth"[MeSH Terms] OR "mouth"[All Fields] OR "oral"[All Fields]) AND "chemotherapy*"[All Fields]) OR (("adjuvancy"[All Fields] OR "adjuvanted"[All Fields] OR "adjuvanting"[All Fields] OR "adjuvants"[All Fields] OR "adjuvants, pharmaceutic"[MeSH Terms] OR ("adjuvants"[All Fields] AND "pharmaceutic"[All Fields]) OR "pharmaceutic adjuvants"[All Fields] OR "adjuvant"[All Fields] OR "adjuvants, immunologic"[MeSH Terms] OR ("adjuvants"[All Fields] AND "immunologic"[All Fields]) OR "immunologic adjuvants"[All Fields] OR "adjuvated"[All Fields] OR "adjuvation"[All Fields] OR "adjuvent"[All Fields]) AND "chemotherapy*"[All Fields]) OR (("adjuvancy"[All Fields] OR "adjuvanted"[All Fields] OR "adjuvanting"[All Fields] OR "adjuvants"[All Fields] OR "adjuvants, pharmaceutic"[MeSH Terms] OR ("adjuvants"[All Fields] AND "pharmaceutic"[All Fields]) OR "pharmaceutic adjuvants"[All Fields] OR "adjuvant"[All Fields] OR "adjuvants, immunologic"[MeSH Terms] OR ("adjuvants"[All Fields] AND "immunologic"[All Fields]) OR "immunologic adjuvants"[All Fields] OR "adjuvated"[All Fields] OR "adjuvation"[All Fields] OR "adjuvent"[All Fields]) AND "Drug"[All Fields] AND "therapy*"[All Fields]) OR "chemotherapy*"[All Fields] OR (("anticancer"[All Fields] OR "anticancers"[All Fields]) AND "medication*"[All Fields]) OR (("anticancer"[All Fields] OR "anticancers"[All Fields]) AND "therapy*"[All Fields]) OR (("anticancer"[All Fields] OR "anticancers"[All Fields]) AND "Drug"[All Fields] AND "treatment*"[All Fields])) AND ("Breast Neoplasms"[All Fields] OR "Breast Neoplasm"[All Fields] OR "neoplasm breast"[All Fields])) OR ("Breast Tumors"[All Fields] OR "Breast Tumor"[All Fields] OR "tumor breast"[All Fields] OR "tumors breast"[All Fields] OR "neoplasms breast"[All Fields] OR "Breast Cancer"[All Fields] OR "cancer breast"[All Fields] OR "Mammary Cancer"[All Fields] OR "cancer mammary"[All Fields] OR "cancers mammary"[All Fields] OR "Mammary Cancers"[All Fields] OR "Malignant Neoplasm of Breast"[All Fields] OR "Breast Malignant Neoplasm"[All Fields] OR "Breast Malignant Neoplasms"[All Fields] OR "Malignant Tumor of Breast"[All Fields] OR "Breast Malignant Tumor"[All Fields] OR "Breast Malignant Tumors"[All Fields] OR "Cancer of Breast"[All Fields] OR "Cancer of the Breast"[All Fields] OR "mammary carcinoma human"[All Fields] OR ("Breast Neoplasms"[MeSH Terms] OR ("breast"[All Fields] AND "neoplasms"[All Fields]) OR "Breast Neoplasms"[All Fields] OR ("carcinoma"[All Fields] AND "human"[All Fields] AND "mammary"[All Fields])) OR ("Breast Neoplasms"[MeSH Terms] OR ("breast"[All Fields] AND "neoplasms"[All Fields]) OR "Breast Neoplasms"[All Fields] OR ("carcinomas"[All Fields] AND "human"[All Fields] AND "mammary"[All Fields])) OR ("Breast Neoplasms"[MeSH Terms] OR ("breast"[All Fields] AND "neoplasms"[All Fields]) OR "Breast Neoplasms"[All Fields] OR ("mammary"[All Fields] AND "carcinomas"[All Fields] AND "human"[All Fields])) OR "Human Mammary Carcinoma"[All Fields] OR ("Breast Neoplasms"[MeSH Terms] OR ("breast"[All Fields] AND "neoplasms"[All Fields]) OR "Breast Neoplasms"[All Fields] OR ("mammary"[All Fields] AND "neoplasms"[All Fields] AND "human"[All Fields])) OR ("Breast Neoplasms"[MeSH Terms] OR ("breast"[All Fields] AND "neoplasms"[All Fields]) OR "Breast Neoplasms"[All Fields] OR ("human"[All Fields] AND "mammary"[All Fields] AND "neoplasm"[All Fields])) OR "Human Mammary Neoplasms"[All Fields] OR ("Breast Neoplasms"[MeSH Terms] OR ("breast"[All Fields] AND "neoplasms"[All Fields]) OR "Breast Neoplasms"[All Fields] OR ("neoplasm"[All Fields] AND "human"[All Fields] AND "mammary"[All Fields])) OR ("Breast Neoplasms"[MeSH Terms] OR ("breast"[All Fields] AND "neoplasms"[All Fields]) OR "Breast Neoplasms"[All Fields] OR ("neoplasms"[All Fields] AND "human"[All Fields] AND "mammary"[All Fields])) OR ("Breast Neoplasms"[MeSH Terms] OR ("breast"[All Fields] AND "neoplasms"[All Fields]) OR "Breast Neoplasms"[All Fields] OR ("mammary"[All Fields] AND "neoplasm"[All Fields] AND "human"[All Fields])) OR "Human Mammary Carcinomas"[All Fields] OR "Breast Carcinoma"[All Fields] OR "Breast Carcinomas"[All Fields] OR "carcinoma breast"[All Fields] OR "carcinomas breast"[All Fields])) AND ("measure*"[All Fields] OR "assess*"[All Fields] OR "evaluat*"[All Fields] OR "method*"[All Fields] OR "instrument*"[All Fields] OR "scale*"[All Fields] OR ("survey s"[All Fields] OR "surveyed"[All Fields] OR "surveying"[All Fields] OR "surveys and questionnaires"[MeSH Terms] OR ("surveys"[All Fields] AND "questionnaires"[All Fields]) OR "surveys and questionnaires"[All Fields] OR "survey"[All Fields] OR "surveys"[All Fields]) OR ("tool s"[All Fields] OR "tools"[All Fields]) OR "questionnaire*"[All Fields]) AND ("womans"[All Fields] OR "women"[MeSH Terms] OR "women"[All Fields] OR "woman"[All Fields] OR "women s"[All Fields] OR "womens"[All Fields] OR ("femal"[All Fields] OR "female"[MeSH Terms] OR "female"[All Fields] OR "females"[All Fields] OR "female s"[All Fields] OR "femals"[All Fields]) OR ("womans"[All Fields] OR "women"[MeSH Terms] OR "women"[All Fields] OR "woman"[All Fields] OR "women s"[All Fields] OR "womens"[All Fields]) OR "Women Groups"[All Fields] OR ("women"[MeSH Terms] OR "women"[All Fields] OR ("women"[All Fields] AND "groups"[All Fields]) OR "Women Groups"[All Fields]) OR "Women's Group"[All Fields] OR ("women"[MeSH Terms] OR "women"[All Fields] OR ("women s"[All Fields] AND "group"[All Fields]) OR "Women's Group"[All Fields]) OR ("girl s"[All Fields] OR "women"[MeSH Terms] OR "women"[All Fields] OR "girls"[All Fields]) OR ("women"[MeSH Terms] OR "women"[All Fields] OR "girl"[All Fields])) AND ("africa"[MeSH Terms] OR "africa"[All Fields] OR "africa s"[All Fields] OR "africas"[All Fields] OR "africa*"[All Fields] OR "Developing countries"[All Fields] OR "saharan*"[All Fields] OR ("algeria"[MeSH Terms] OR "algeria"[All Fields]) OR ("angola"[MeSH Terms] OR "angola"[All Fields] OR "angola s"[All Fields]) OR ("benin"[MeSH Terms] OR "benin"[All Fields] OR "benin s"[All Fields]) OR ("botswana"[MeSH Terms] OR "botswana"[All Fields] OR "botswana s"[All Fields]) OR "Burkina Faso"[All Fields] OR ("burundi"[MeSH Terms] OR "burundi"[All Fields]) OR "Cabo Verde"[All Fields] OR ("cameroon"[MeSH Terms] OR "cameroon"[All Fields] OR "cameroons"[All Fields] OR "cameroon s"[All Fields]) OR "Central African Republic"[All Fields] OR ("chad"[MeSH Terms] OR "chad"[All Fields]) OR ("comoros"[MeSH Terms] OR "comoros"[All Fields] OR "comoro"[All Fields]) OR "Democratic Republic of the Congo"[All Fields] OR "Republic of the Congo"[All Fields] OR "Cote d'Ivoire"[All Fields] OR ("djibouti"[MeSH Terms] OR "djibouti"[All Fields]) OR ("egypt"[MeSH Terms] OR "egypt"[All Fields] OR "egypt s"[All Fields]) OR "Equatorial Guinea"[All Fields] OR ("eritrea"[MeSH Terms] OR "eritrea"[All Fields]) OR ("eswatini"[MeSH Terms] OR "eswatini"[All Fields]) OR ("eswatini"[MeSH Terms] OR "eswatini"[All Fields] OR "swaziland"[All Fields]) OR ("ethiopia"[MeSH Terms] OR "ethiopia"[All Fields] OR "ethiopia s"[All Fields]) OR ("gabon"[MeSH Terms] OR "gabon"[All Fields]) OR ("gambia"[MeSH Terms] OR "gambia"[All Fields] OR "gambia s"[All Fields]) OR ("ghana"[MeSH Terms] OR "ghana"[All Fields] OR "ghana s"[All Fields]) OR ("guinea"[MeSH Terms] OR "guinea"[All Fields] OR "guinea s"[All Fields] OR "guineas"[All Fields]) OR "Guinea-Bissau"[All Fields] OR ("kenya"[MeSH Terms] OR "kenya"[All Fields] OR "kenya s"[All Fields]) OR ("lesotho"[MeSH Terms] OR "lesotho"[All Fields] OR "lesotho s"[All Fields]) OR ("liberia"[MeSH Terms] OR "liberia"[All Fields] OR "liberia s"[All Fields]) OR ("libya"[MeSH Terms] OR "libya"[All Fields]) OR ("madagascar"[MeSH Terms] OR "madagascar"[All Fields] OR "madagascar s"[All Fields]) OR ("malawi"[MeSH Terms] OR "malawi"[All Fields] OR "malawi s"[All Fields]) OR ("mali"[MeSH Terms] OR "mali"[All Fields]) OR ("mauritania"[MeSH Terms] OR "mauritania"[All Fields]) OR ("mauritius"[MeSH Terms] OR "mauritius"[All Fields]) OR ("morocco"[MeSH Terms] OR "morocco"[All Fields]) OR ("mozambique"[MeSH Terms] OR "mozambique"[All Fields] OR "mozambique s"[All Fields]) OR ("namibia"[MeSH Terms] OR "namibia"[All Fields] OR "namibia s"[All Fields]) OR ("niger"[MeSH Terms] OR "niger"[All Fields]) OR ("nigeria"[MeSH Terms] OR "nigeria"[All Fields] OR "nigeria s"[All Fields]) OR ("rwanda"[MeSH Terms] OR "rwanda"[All Fields] OR "rwanda s"[All Fields]) OR "Sao Tome and Principe"[All Fields] OR ("senegal"[MeSH Terms] OR "senegal"[All Fields] OR "senegal s"[All Fields]) OR ("seychelles"[MeSH Terms] OR "seychelles"[All Fields]) OR "Sierra Leone"[All Fields] OR ("somalia"[MeSH Terms] OR "somalia"[All Fields] OR "somalia s"[All Fields]) OR "South Africa"[All Fields] OR "South Sudan"[All Fields] OR ("sudan"[MeSH Terms] OR "sudan"[All Fields] OR "sudans"[All Fields] OR "sudan s"[All Fields]) OR ("tanzania"[MeSH Terms] OR "tanzania"[All Fields] OR "tanzania s"[All Fields]) OR ("togo"[MeSH Terms] OR "togo"[All Fields]) OR ("tunisia"[MeSH Terms] OR "tunisia"[All Fields]) OR ("uganda"[MeSH Terms] OR "uganda"[All Fields] OR "uganda s"[All Fields]) OR ("zambia"[MeSH Terms] OR "zambia"[All Fields] OR "zambia s"[All Fields]) OR ("zimbabwe"[MeSH Terms] OR "zimbabwe"[All Fields] OR "zimbabwe s"[All Fields]))) AND ((humans[Filter]) AND (female[Filter]) AND (1990/1/1:2025/3/9[pdat]) AND (english[Filter]))

8,360 11:43:07

……………………………………………………………………………………………………

**Google Scholar search**

Search Terms

"breast cancer" and ("medication adherence" or adhere*) and ("anticancer agents" OR Chemotherapy) and ("measures" or method*) and women and Africa*

- [Custom range...](javascript:void(0))

 —

……………………………………………………………………………………………………

**EBSCOHOST SEARCH**

Search Terms

"medication adherence" OR "medication compliance" OR "medication nonadherence" OR "medication noncompliance" OR Adhere* OR Complian* AND "Administration, oral" OR Antineoplastic agents OR chemotherapy* AND "breast neoplasms" OR "breast cancer" OR breast tumor* OR "breast carcinoma" AND women OR female OR Girl* AND method* OR measure* OR assess* OR instrument OR Scale* AND Africa OR African countries OR Africa*

Searching:  Health Source: Nursing/Academic Edition (and 1 more)

Full Text Peer Reviewed

01/01/1990 - 03/09/2025

01/01/1990 - 03/09/2025

Academic Journals

[Advanced search](https://research-ebsco-com.ukzn.idm.oclc.org/c/rv2oto/search/advanced/search-options).
